# Application of *Mulberry nigra* to absorb heavy metal, mercury, from the environment of green space city

**DOI:** 10.1016/j.toxrep.2018.05.006

**Published:** 2018-05-21

**Authors:** Seyed Armin Hashemi, Sahar Tabibian

**Affiliations:** a Department of Forestry, Lahijan Branch, Islamic Azad University, Lahijan, Iran; b Department of Agriculture and natural resources, Payame Noor University, P.OBox, 19395-3697, Tehran, Iran

**Keywords:** *Mulberry nigra*, Mercury, Heavy metals, North of Iran

## Abstract

Phytoremediation is one of the methods for Bioremediation of the soils which has been noticed in recent decades. Two years sapling of *Mulberry nigra* selected and Mercury(II) nitrate solution with 30, 50 and 70 mg/L concentrations, after add solutions in to soil of sapling *Mulberry nigra*, eight months’ later leaf, stem and roots were selected and Measuring mercury was determined by Atomic Absorption Spectrometer. The maximum level of mercury accumulation in the leaf, stem and root was 55.67, 50 and 65 mg/kg, respectively. Mercury could be absorbed easily by the plant’s root and accumulated in the plants.

## Highlights

• The obtained results from the research suggest that aerial organs of *Mulberry nigra* have had significant difference for mercury accumulation in various concentrations.
• According to the results, *Mulberry nigra* species, seems to be an appropriate species for refining soils contaminated by mercury.
• Mercury pollution is most pollution in soil of north of Iran in industrial park area.
• Studies in environment heavy metal pollution in soil of urban forestry in most important for human health.
• *Mulbery nigra* is a fast-growing species from medium to high trees, is resistant towards various soils and prefers visors to shade-friendly sites .In this research it was studied that how much a *Mulbery nigra* could absorb the mercury from the environment.

## Graphical abstract

**Figure:**
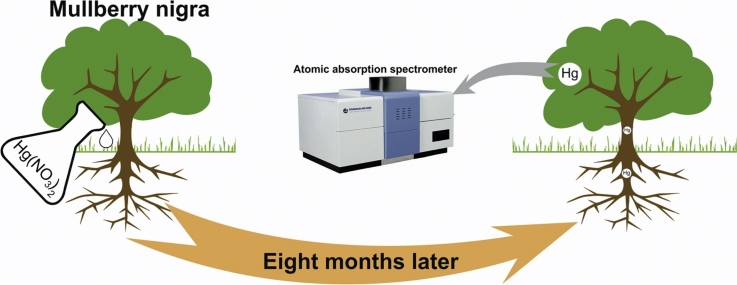

## 1 Introduction

Human sources for releasing the mercury to the environment is by secondary products of various industrial processes like coal burning, combustion of fossil fuels, mercurial vapor lamps and producing Chloralkali [1]. Phytoremediation is one of the methods for Bioremediation of the soils which has been noticed in recent decades. In this method, resistant plants are used for refining the soils contaminated by organic and inorganic components. The advantages of this method among the others are its simplicity, inexpensiveness and exploiting possibility in a broad level. Selection of plant has a great importance in this method. Plant selection depends on climate conditions and also the level of pollution [2,3]. The mercury is toxic and is absorbed easily by respiratory system and damages Stomach and intestines. Existence of this element in the air is harmful. By initiation of industrial era, the level of mercury has been increased significantly. The mercury has harmful effect of the human beings and wildlife as well as various mediums and food (especially fish) all around the world [4]. The level of entered mercury into the environment has increased from the beginning of industrial era. The mercury contamination is first due to man activities from which 60–90% of total mercury released from man activities is caused by industrial activities in 1995–1999. Mercury emission caused by man activities in Asia has increased from about 30% of total universal emission to 56% [5,6,7].

The most important way for mercury emission is its emission in the air. However, the mercury is also released from various sources in the water and earth. In the case of releasing, the mercury is remained in various forms and circulates among the air, water, deposits, soil and plants. The study of heavy metals’ distribution in the dusts of the streets and the soil of industrial towns’ workshops in Jordan suggested that heavy metal density is more accumulated in surface soil and it is decreasing in the lower level of the soil. The man and industrial activities in the town workshops are possible sources for accumulating the heavy metals of Zn, Cu, Ni and Pb from which a significant portion was related to industrial sources in the town [8].

*Mulbery nigra* is a fast-growing species from medium to high trees, is resistant towards various soils and prefers visors to shade-friendly sites. In this research it was studied that how much a *Mulbery nigra* could absorb the mercury from the environment.

## 2 Method and materials

Two years saplings were transited into greenhouse and were maintained there for 20 days in order to adapt to new condition. Non-contaminated soil (applied natural soil in this analysis) was provided from 0 to 30 depth of one of plantations. Then they were dried and passed from a 2 mm sieve.in order to provide a soil contaminated by Mercury(II) nitrate, spraying on the soil by 30, 50 and 70 mg density was used in this study and the required amount of the solution was sprayed on the soil gradually, then it was contaminated by the soil uniformly and the vases were filled by them. The seedlings with the same age and size were chosen in sufficient number and were planted in the vase. Afterward, the vases were maintained in the greenhouse and the soil moisture was kept in the capacity level by weighted method. Watering was done by distilled water when requiring and eight months after seedlings growth, the aerial organs were harvested and washed by water, then they were dried in the oven by 70 °C. The level of mercury in plant samples was determined by dry digestion method after samples digestion with atomic absorption spectrometry. The obtained data from plants tests were organized in SPSS software. In order to analyze data for determining metal accumulation level in aerial organs and the root of plant, ANOVA test and in order to compare the effect of mercury concentration on the leaf, stem and root, Duncan test were used.

## 3 Results

**Fig. 1:**
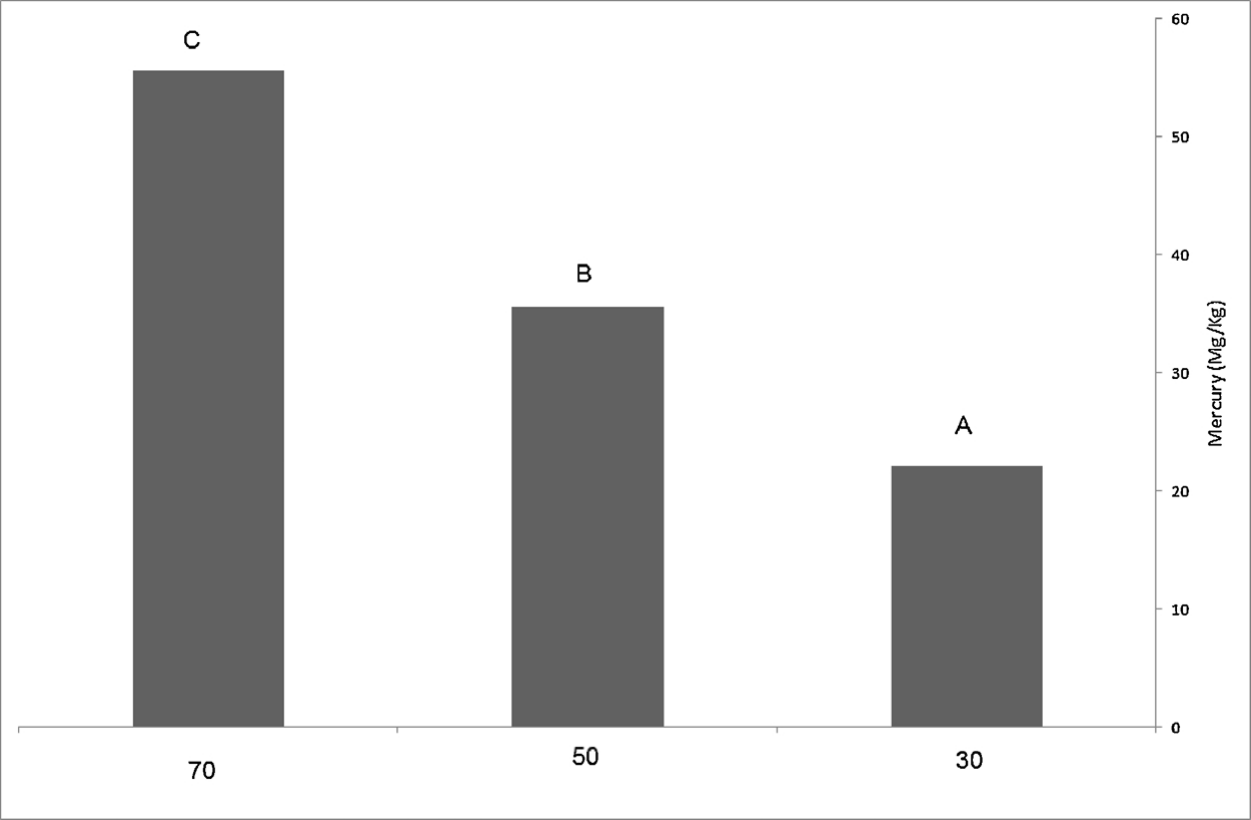
The mean of mercury absorption in aerial organ of leaf in *Mulberry nigra* sapling.

Comparison of mercury level among the studied concentrations, using Duncan test suggested that there is significant difference in accumulation level of mercury among 30, 50 and 70 mg/l concentrations in probability level of 95%. Such that the highest level of mercury accumulation was 55.67 mg/kg and the lowest level was 22.2 mg/kg in 20 mg/l density (Fig. 1).

**Fig. 2:**
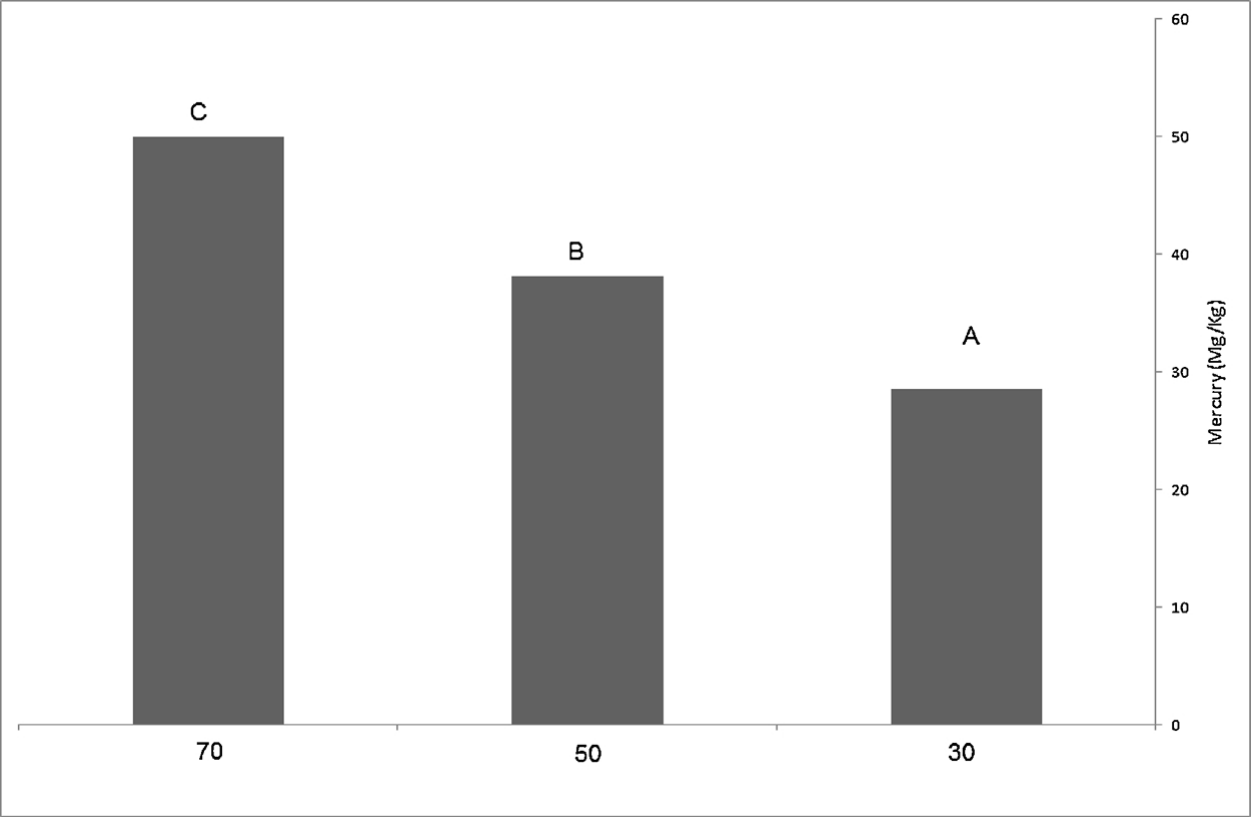
The mean of mercury absorption in the stem of *Mulberry nigra* sapling.

The comparison of mercury among studied concentrations using Duncan test suggested that there is significant difference among 30, 50,70 mg/L by 95% probability in accumulation level of mercury in the stem m so that the highest level of Mercury accumulation is 50 mg/kg and the lowest level was 28.6 mg/kg (Fig. 2).

**Fig. 3:**
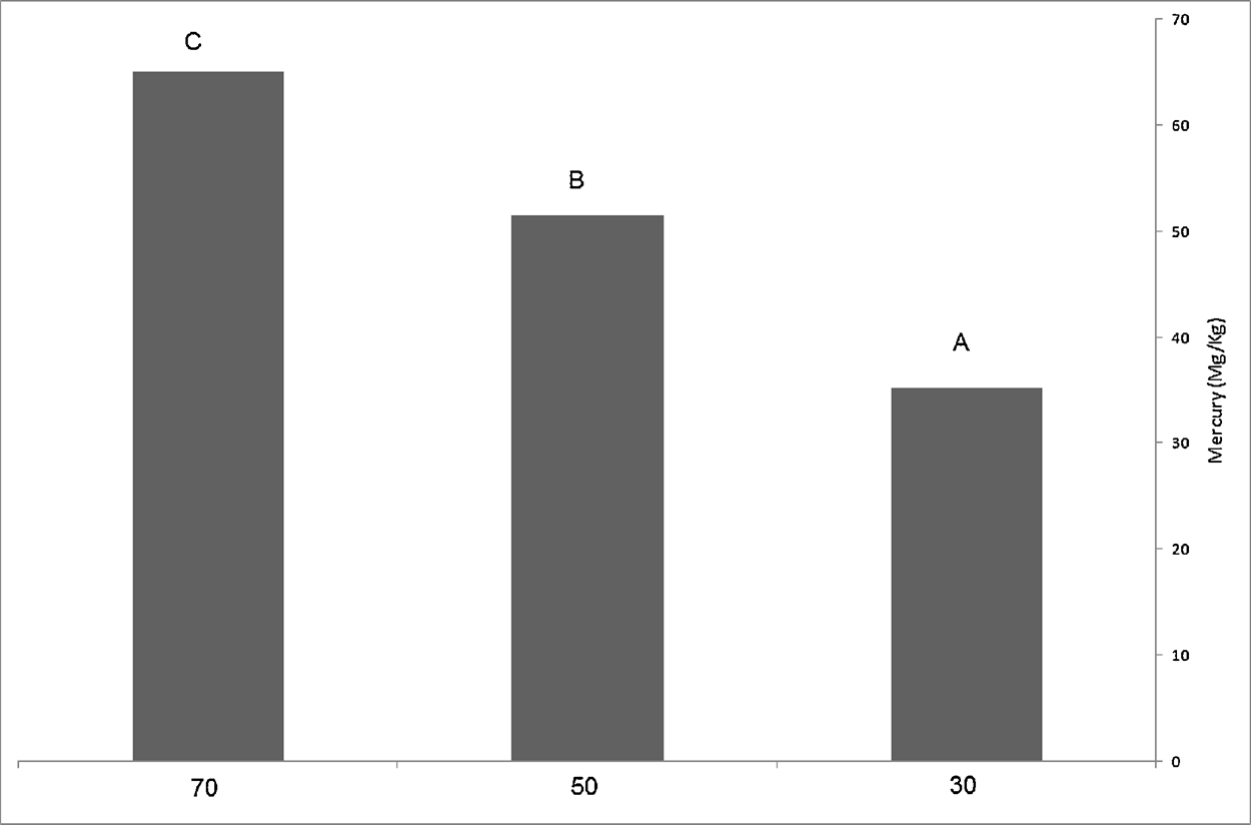
The mean of mercury accumulation in the root of *Mulberry nigra* sapling.

Comparison of the mercury means among the studied concentrations, using Duncan test suggested that there is significant difference among 30, 50, and 70 mg/L by 95% probability in mercury accumulation. Such that the highest level of mercury accumulation is 65 mg/kg and the lowest level of mercury accumulation is 35.3 mg/kg (Fig. 3).

## 4 Discussion

The obtained results from the research suggest that aerial organs of *Mulberry nigra* have had significant difference for mercury accumulation in various concentrations. By increasing the concentration of soil mercury, the amount of its accumulation in aerial organs of leaf, stem of *Mulberry nigra* has increased as well. By studying the effect of mercury on some physiology parameters in eucalyptus and also comparing the accumulation and mercury transition in this study suggested that the absorption of this metal in the root is more than it in leaf and stem [9,10]. About mercury accumulation in *Acer velutinum*, the results suggest that various contamination densities have been effective and by increasing mercury, accumulation level in the root is increased as well (Fig. 2) concluded in their research that although the mercury is not a nutrient, could be absorbed easily by the plant’s root and accumulated in the plants with some concentrations which is harmful for food chain.

## 5 Conclusion

The factors of stems, roots, soil, are a significant difference in terms of mercury accumulation and this significant difference shows the amount of mercury absorption in *Mullberry nigra* tree species and the ability of *Mullberry nigra* in the amount of contamination accumulation is appropriate. Study revealed that concentrations of lead, zinc, mercury, cadmium and nickel were measured in the leaf of seven species of deciduous trees (Indian horse-chestnut, maple, *Acer cappadocicumGled*, ash, *Platanus orientalis*, poplar and acacia) in urban areas which the highest accumulation amount of cadmium and zinc, lead, mercury and nickel was in poplar, Indian horse-chestnut and acacia, and acacia and ash, respectively [11,12]. The mercury accumulation in plant tissues in cell surface also may be toxic and cause growth decrease. Therefore, preventing mercury absorption by plant’s roots, could be a strong strategy for minimizing biologic side effects of this element. In phytoremediation topics of heavy metals, tolerance factors of the plant against metals, plants’ root system, and the capability of transition from underground organs to aerial organs (transition factor), growth speed and high biomass must be considered. In this research, according to mentioned points, *Mulberry nigra* is an appropriate species for refining soils contaminated by mercury.

## Transparency document

Transparency document
